# Metabolic Dysregulation and Metabolite-Targeted Interventions in Autism Spectrum Disorder: Mechanisms and Translational Perspectives

**DOI:** 10.3390/metabo16100749

**Published:** 2026-10-05

**Authors:** Yu Fang, Zechen Dou, Yanan Liu, Yishan Zong, Shengxiang Wang, Shangyong Li, Ningning He, Ning Li

**Affiliations:** 1 School of Basic Medicine, Qingdao Medical College, Qingdao University, Qingdao 266000, Chinadouzechen@qdu.edu.cn (Z.D.);; 2 Jinan Institute of Child Health Care, Children’s Hospital Affiliated to Shandong University (Jinan Children’s Hospital), Jing-shi Road 23976, Jinan 250022, China

**Keywords:** autism spectrum disorder, metabolism, metabolomics, metabolite-targeted interventions, gut–brain axis, clinical translation

## Abstract

Autism spectrum disorder (ASD) is a heterogeneous neurodevelopmental disorder marked by impaired social communication and restricted repetitive behaviors. Metabolic dysregulation is increasingly recognized as a key intermediate mechanism linking genetic and environmental factors to abnormal neurodevelopment in ASD. This review summarizes ASD-related metabolic abnormalities involving gut microbial metabolites, energy metabolism, neurotransmitter pathways, and immune–metabolic crosstalk. Such metabolic disturbances affect neurodevelopment via immune activation, oxidative stress, mitochondrial dysfunction, and disrupted neurotransmitter homeostasis, ultimately impairing synaptic plasticity and neural connectivity. We also outline the preclinical and clinical progress of metabolite-targeted interventions, which show potential to modulate relevant metabolic pathways. However, it should be noted that improvements in specific metabolic biomarkers may only be associated with, rather than definitively drive, behavioral changes. However, current studies are constrained by inconsistent trial designs, small cohorts, and inadequate mechanistic verification; furthermore, interindividual metabolic heterogeneity limits universal intervention efficacy. We discuss clinical translation barriers and propose future priorities: defining metabolic subtypes, validating biomarkers, and developing precision metabolic interventions. Elucidating these metabolic mechanisms will advance targeted, personalized therapies to improve neurodevelopmental outcomes in ASD.

## 1. Introduction

Autism spectrum disorder (ASD) is a highly heterogeneous neurodevelopmental disorder characterized by deficits in social communication and interaction, repetitive and stereotyped behaviors, and restricted interests [1,2]. According to the 2025 report of the US Centers for Disease Control and Prevention (CDC), the prevalence of ASD among 8-year-old children in 2022 was 1 in 31 [3], while systematic global epidemiological data indicate a median prevalence of approximately 1% [4]. Current standard therapies rely primarily on behavioral interventions and symptomatic management. While these approaches offer partial relief, they fail to target the underlying biological mechanisms, presenting a substantial clinical bottleneck. As an important manifestation of gene–environment interactions, metabolic dysregulation is emerging as a critical nexus for elucidating the pathogenesis of ASD [5,6].

Accumulating evidence suggests that the onset of ASD results from the combined effects of genetic susceptibility and environmental exposures, with metabolic disorders acting as a key convergent pathway that influences neurodevelopmental trajectories [7,8,9]. Currently, four core areas of metabolic abnormalities have been reported in ASD cohorts: gut microbiota-derived metabolic shifts, energy metabolism deficits, neurotransmitter dysregulation, and immune–metabolic interactions [10]. These metabolic disturbances may cross-link and act synergistically, ultimately mediating abnormal neural development and the core phenotypes of ASD. However, existing reviews in this field mostly focus on the phenotypic characterization of these metabolic abnormalities, lacking a systematic integration of metabolite-targeted interventions from a translational perspective. Specifically, two key research gaps remain: First, the logical chain of “targetable metabolic abnormalities-intervention mechanisms-clinical efficacy” has not been systematically mapped to correspond intervention strategies with core metabolic targets. Second, the core barriers to clinical translation have not been systematically dissected, and feasible precision clinical trial designs based on metabolic subtyping are still lacking. While several recent excellent reviews have comprehensively detailed the phenotypic characterization of metabolic abnormalities and gut microbiome shifts in ASD [6,10,11], our work provides a distinct perspective. Unlike the previous literature, this review aims to bridge these gaps by systematically mapping the logical chain of “targetable metabolic abnormalities-intervention mechanisms-clinical efficacy” and proposing a structured clinical translational roadmap utilizing a GRADE-informed priority classification.

Given the potential hub role of metabolic dysregulation in ASD pathology, metabolite-targeted interventions represent a promising avenue to overcome the limitations of traditional treatments [12,13]. Currently, several intervention strategies show considerable translational potential: Clinically, agents such as AB-2004 [14], the ketogenic diet [15], and probiotic preparations [16] have been reported to alleviate specific ASD symptoms. In preclinical models, interventions with metabolites such as acetyl-L-carnitine [17] and indolepropionic acid [18] have demonstrated the ability to reverse autistic-like behaviors.

This review systematically integrates the existing evidence on ASD metabolic abnormalities, focusing on the four core metabolic modules and the potential mechanisms by which they mediate abnormal neural development. We summarize the research progress and existing limitations of metabolite-targeted interventions, dissect the barriers to clinical translation, and explore future research priorities. The aim is to provide a comprehensive theoretical framework for understanding ASD pathogenesis and offer actionable insights for the clinical translation of targeted therapies.

As a narrative review, our literature search was conducted using the PubMed database. The search strategy utilized combinations of keywords such as ‘autism spectrum disorder’, ‘metabolism’, ‘metabolomics’, ‘metabolite-targeted interventions’, and ‘gut–brain axis’. It should be noted that rather than aiming for an exhaustive systematic extraction of all available studies, articles were selectively included based on their clinical relevance to mapping the targeted metabolic pathways and exploring intervention mechanisms.

## 2. Metabolic Perspective on ASD Pathogenesis

### 2.1. The Core Pathophysiological Cascade

Before detailing specific pathways, it is crucial to emphasize that the metabolic abnormalities currently observed in ASD primarily reflect correlations rather than definitively confirmed causations. These metabolic alterations may be a contributing cause of the disorder, a secondary consequence of the condition itself, or merely a byproduct of comorbid factors (such as gastrointestinal symptoms or highly restricted dietary habits). A comprehensive elucidation of the precise causal mechanisms remains a prerequisite for future clinical trials, and the pathways discussed herein should be interpreted as potential associations.

**Figure 1:**
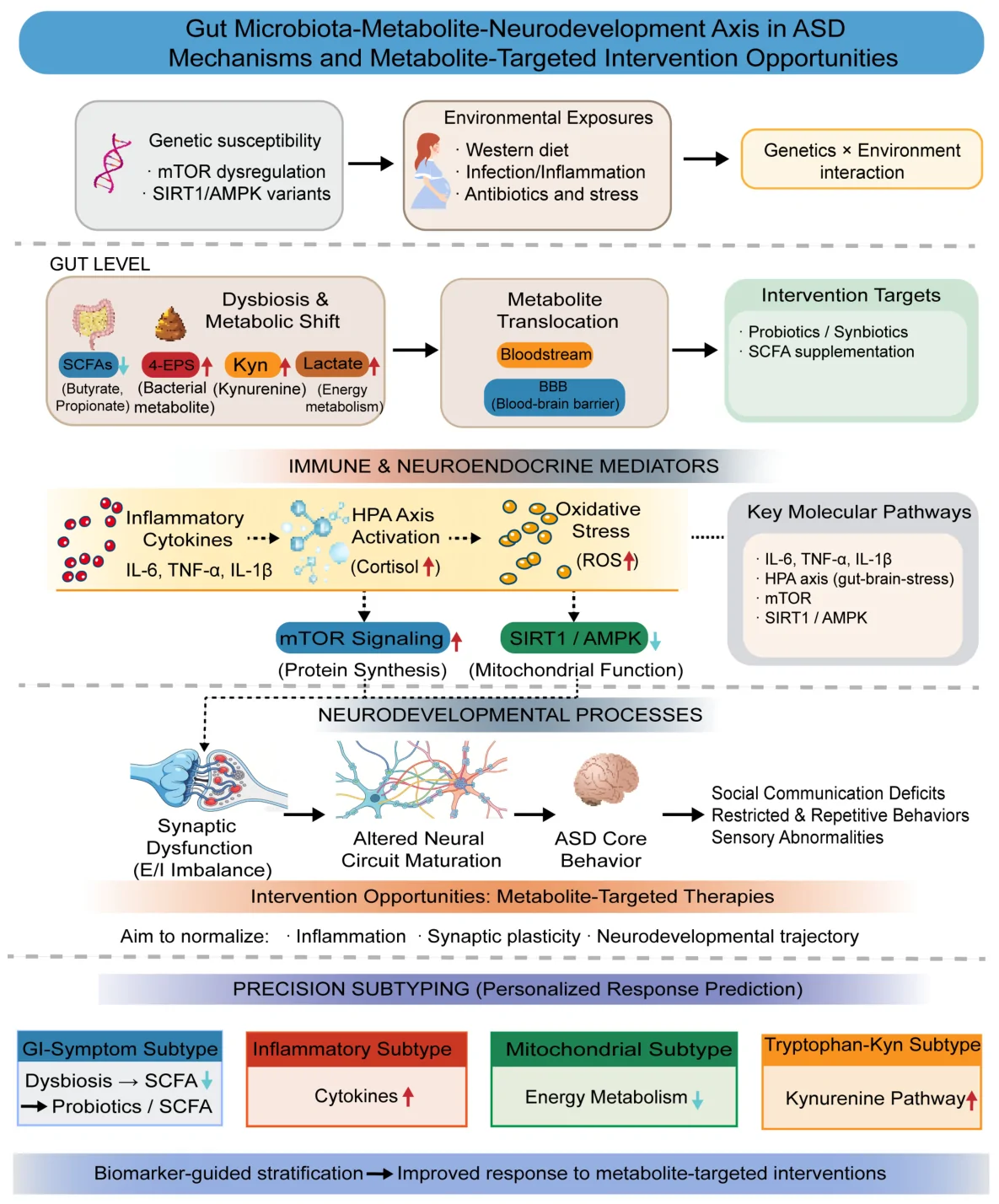
The gut microbiota-metabolite-neurodevelopment axis in ASD: mechanisms and metabolite-targeted intervention opportunities. This schematic conceptualizes how genetic susceptibility and environmental exposures may interact to contribute to ASD pathogenesis via the gut–brain axis. Gut microbiota dysbiosis and associated metabolic shifts (involving SCFAs, 4-EPS, Kyn, and lactate) are concurrently associated with systemic inflammation, oxidative stress, and the dysregulation of the HPA axis, mTOR, and SIRT1/AMPK pathways. These parallel cascades are hypothesized to exacerbate abnormal synaptic development (E/I imbalance), altered neural circuit maturation, and core ASD behavioral phenotypes. The figure also highlights potential targets for metabolite-based therapies and proposes biomarker-guided metabolic subtypes for precision response prediction. Note: Solid lines represent pathways with established macroscopic evidence, whereas dashed lines denote proposed hypothetical mechanisms and parallel signaling cascades. Elements depicted as precision subtyping constitute a conceptual model based on the current metabolic literature rather than definitively confirmed clinical guidelines. The upward and downward arrows (↑/↓) denote an increase and decrease, respectively. Abbreviations: ASD = autism spectrum disorder; SCFAs = short-chain fatty acids; 4-EPS = 4-ethylphenyl sulfate; Kyn = kynurenine; BBB = blood–brain barrier; mTOR = mechanistic target of rapamycin; SIRT1 = sirtuin 1; AMPK = AMP-activated protein kinase; HPA = hypothalamic–pituitary–adrenal; ROS = reactive oxygen species; E/I = excitatory/inhibitory.

Metabolic dysregulation may represent a convergent pathway through which genetic susceptibility and environmental factors interact to influence neurodevelopment [19]. Genetic factors primarily mediate this pathological process by altering the activity of metabolic enzymes or disrupting the homeostasis of metabolic pathways [20]. Complex pathophysiological features frequently associated with ASD—such as mitochondrial deficits, neuroinflammation, oxidative stress, and gut–brain axis dysregulation—are inherently coupled with these metabolic shifts [21,22,23,24,25,26]. It is important to note that the directional causality of these mechanisms remains a subject of ongoing debate. While studies have demonstrated that microbial metabolites can directly modulate host neurodevelopment [27], large-cohort analyses suggest that certain microbiome alterations may manifest as a secondary consequence of ASD-related dietary restrictions rather than acting as primary disease drivers [23]. These diverse mechanisms do not exist in isolation; rather, they interact dynamically within a broader metabolic network. Together, they integrate innate genetic predispositions with acquired environmental stimuli, ultimately influencing neurodevelopmental trajectories and contributing to the core clinical phenotypes of ASD (Figure 1).

Once central nervous system homeostasis is disrupted, environmental and metabolic disturbances are hypothesized to influence neurodevelopment through several converging signaling cascades. For instance, metabolic stress and systemic inflammation may activate the hypothalamic–pituitary–adrenal (HPA) axis, leading to abnormal cortisol fluctuations [28]. Furthermore, metabolic dysregulation can inhibit the SIRT1/AMPK pathway, a critical sensor of cellular energy, and subsequently contribute to the aberrant overactivation of the mammalian target of rapamycin (mTOR) signaling pathway [29]. Such dysregulated energy and nutrient sensing cascades can destabilize synaptic homeostasis, ultimately jeopardizing normal neurodevelopmental trajectories.

### 2.2. Metabolic Alterations in ASD

The metabolic abnormalities in children with ASD mainly involve four metabolic pathways: gut microbiota-derived metabolism, energy metabolism, neurotransmitter metabolism, and immune–metabolic interactions (Table 1).

#### 2.2.1. Gut-Derived Metabolic Alterations

The gut–brain axis represents a bidirectional communication system through which microbial metabolites influence neurodevelopment and neural signaling pathways [22,44]. Alterations in gut microbiota composition and intestinal permeability in ASD are associated with the abnormal production and systemic distribution of microbial metabolites [11].

Several metabolomic studies have reported altered tryptophan and purine metabolites in urine samples from children with ASD compared to neurotypical controls [45,46,47,48]. Pioneering research by the Sarkis K. Mazmanian group at the California Institute of Technology demonstrated elevated systemic levels of 4-EPS in murine models of neurodevelopmental disorders. This gut-derived metabolite traverses the blood–brain barrier and localizes within cerebral regions, where it disrupts oligodendrocyte function and neuronal myelination patterns, thereby exacerbating anxiety-like behaviors in mice. The study further delineated a mechanistic pathway wherein 4-EPS impairs oligodendrocyte precursor cell differentiation via the inhibition of histone deacetylase 3 (HDAC3), resulting in the hypomyelination of prefrontal–limbic circuits [49].

Compounds such as 4-EPS and indole derivatives have been reported to be elevated in multiple ASD cohorts and are associated with specific behavioral phenotypes and gastrointestinal comorbidities [30,50]. However, further stratified analyses revealed that 4-EPS elevation was primarily observed in the subset of children with ASD who had concurrent severe gastrointestinal symptoms, but not in those without such comorbidities. This suggests that the previously reported association may be confounded by gastrointestinal dysfunction rather than acting as a universal core pathological marker of ASD [30].

#### 2.2.2. Energy Metabolism Abnormalities

Disruptions in energy metabolism, particularly mitochondrial dysfunction, are frequently observed in ASD [6,51,52]. Abnormal levels of lactate, pyruvate, ATP, and acylcarnitines suggest underlying impairments in oxidative phosphorylation and fatty acid metabolism.

**Figure 2:**
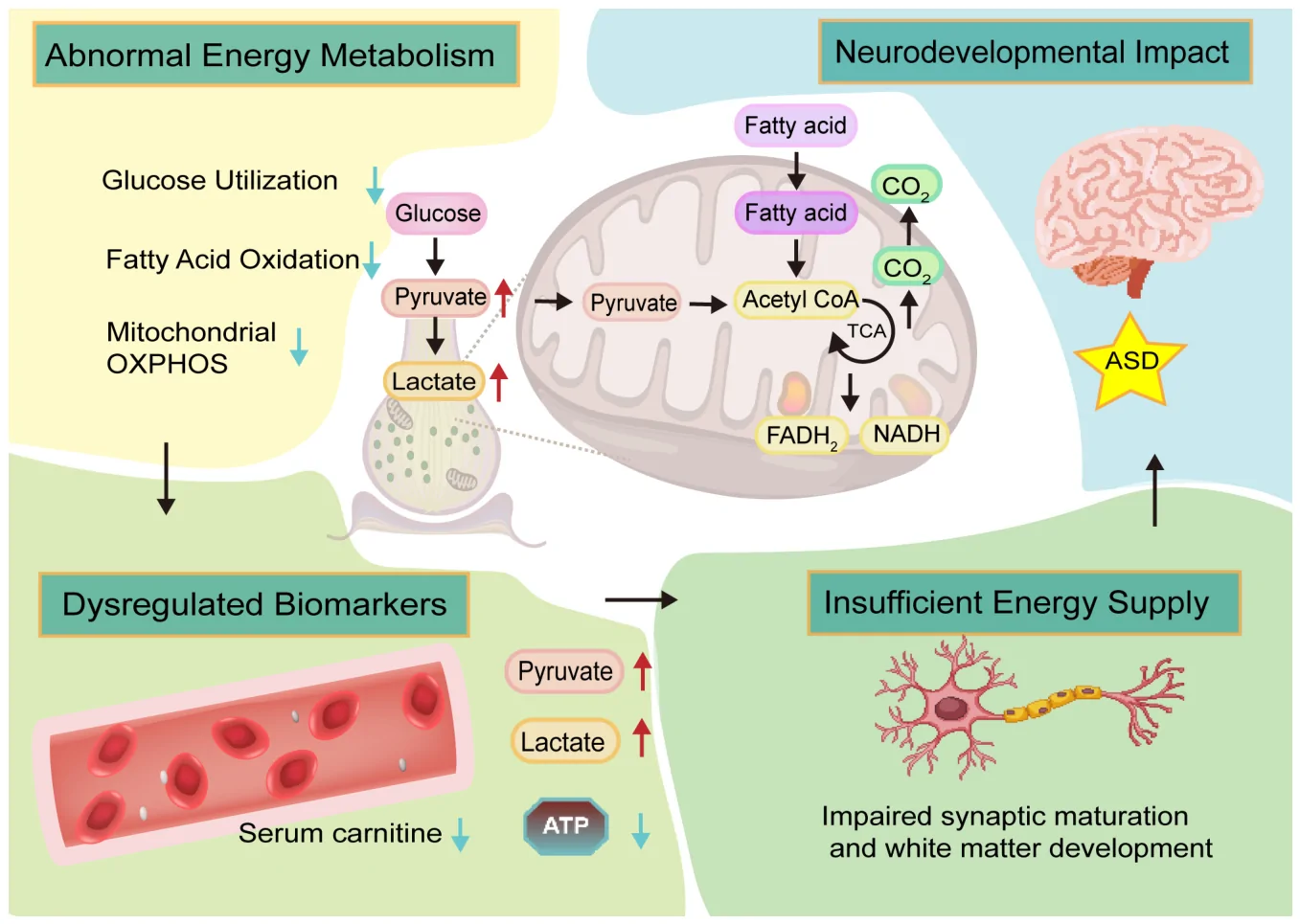
Abnormal energy metabolism in ASD. This schematic depicts the core defects of energy production pathways in ASD, including impaired glucose utilization, fatty acid oxidation, and mitochondrial oxidative phosphorylation (OXPHOS). These metabolic abnormalities lead to consistent dysregulation of clinical biomarkers (elevated pyruvate and lactate, decreased serum carnitine and ATP), resulting in insufficient neuronal energy supply, impaired synaptic maturation, and altered white matter development, which collectively contribute to the core pathophysiology of ASD. The upward and downward arrows (↑/↓) denote an increase and decrease, respectively. Abbreviations: ASD = autism spectrum disorder; OXPHOS = oxidative phosphorylation; ATP = adenosine triphosphate; TCA = tricarboxylic acid.

Several studies have consistently reported abnormalities in metabolic markers—such as elevated lactate and pyruvate, alongside reduced ATP levels—in individuals with ASD [6,26,53,54,55] (Figure 2). In peripheral blood, serum carnitine and acylcarnitine metabolite profiles have shown promise in accurately classifying children with ASD under 5 years of age [37,52,56,57,58,59,60]. Furthermore, metabolomic profiling of post-mortem human brain tissue has identified specific central markers; for instance, the combination of 9-hexadecenoyl carnitine and specific phosphatidylcholines in cerebellar tissue can distinguish ASD from control brains with 80% sensitivity and specificity, providing direct evidence of altered lipid and mitochondrial metabolism in the central nervous system [61].

#### 2.2.3. Neurotransmitter-Related Metabolic Changes

Neurotransmitter homeostasis in ASD is intrinsically linked to metabolic pathways, particularly tryptophan metabolism [52,62]. Dysregulation across the serotonin, kynurenine, and indole pathways has been well documented and is correlated with imbalances in neuroactive metabolites [63,64,65].

**Figure 3:**
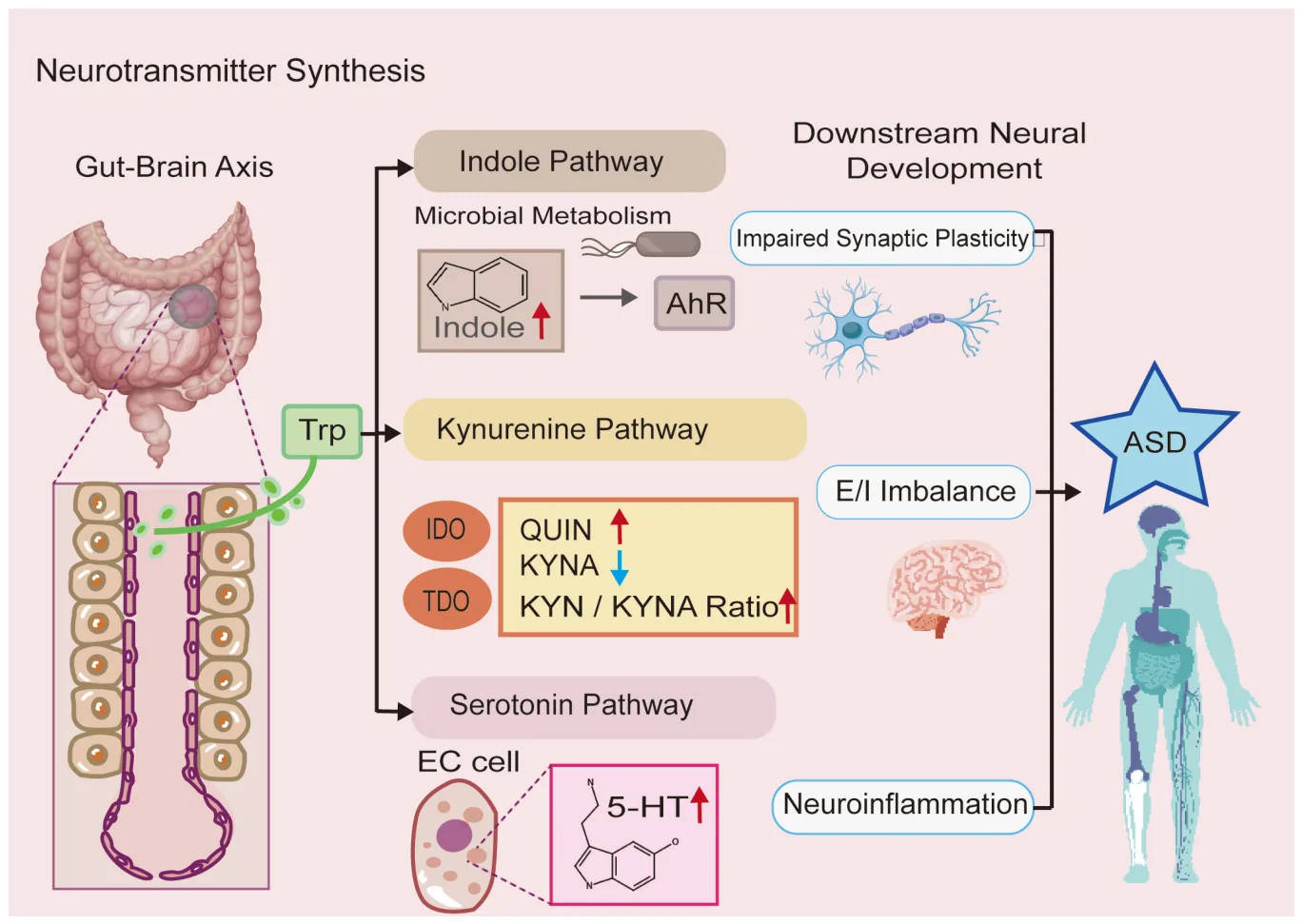
Tryptophan metabolism and neurotransmitter synthesis dysregulation via the gut–brain axis in ASD. This schematic depicts the three core branches of tryptophan (Trp) metabolism (the indole, kynurenine, and serotonin/5-HT pathways) regulated by the gut–brain axis, and their abnormalities in ASD. Dysregulated Trp metabolism leads to altered neurotransmitter production, disrupted synaptic plasticity, excitatory/inhibitory (E/I) imbalance, and excessive neuroinflammation, which contribute to the core neurodevelopmental deficits of ASD. These pathways represent key actionable targets for metabolite-based ASD interventions. The upward and downward arrows (↑/↓) denote an increase and decrease, respectively. Abbreviations: ASD = autism spectrum disorder; Trp = tryptophan; 5-HT = 5-hydroxytryptamine; AhR = aryl hydrocarbon receptor; IDO = indoleamine 2,3-dioxygenase; TDO = tryptophan 2,3-dioxygenase; QUIN = quinolinic acid; KYNA = kynurenic acid; EC = enterochromaffin; E/I = excitatory/inhibitory.

Multiple studies have clarified these neurotransmitter-related metabolic abnormalities in ASD: Hyperserotonemia—defined as elevated whole-blood serotonin levels—has been reported in approximately 30% of individuals with ASD [38]. In pediatric ASD cohorts, specific metabolic profiling reveals that neuroprotective kynurenic acid levels are often decreased while excitotoxic quinolinic acid is elevated [48,66]. While recent large-scale meta-analyses indicate that baseline peripheral blood kynurenine markers may not be universally altered, these urinary and localized metabolic shifts strongly indicate a pronounced dysregulation within the tryptophan-kynurenine pathway [66]. Furthermore, gut microbiome-driven disruption of the indole metabolic pathway may alter tryptophan availability, preferentially shunting it away from serotonin and melatonin synthesis, which ultimately disturbs global neurotransmitter homeostasis [48] (Figure 3).

#### 2.2.4. Immune–Metabolic Interactions

Abnormal immune–metabolic interactions, encompassing maternal immune activation, oxidative stress, and neuroinflammation, play a pivotal role in ASD. These processes are closely linked to systemic metabolic disturbances and may synergistically disrupt neurodevelopment [67,68].

**Figure 4:**
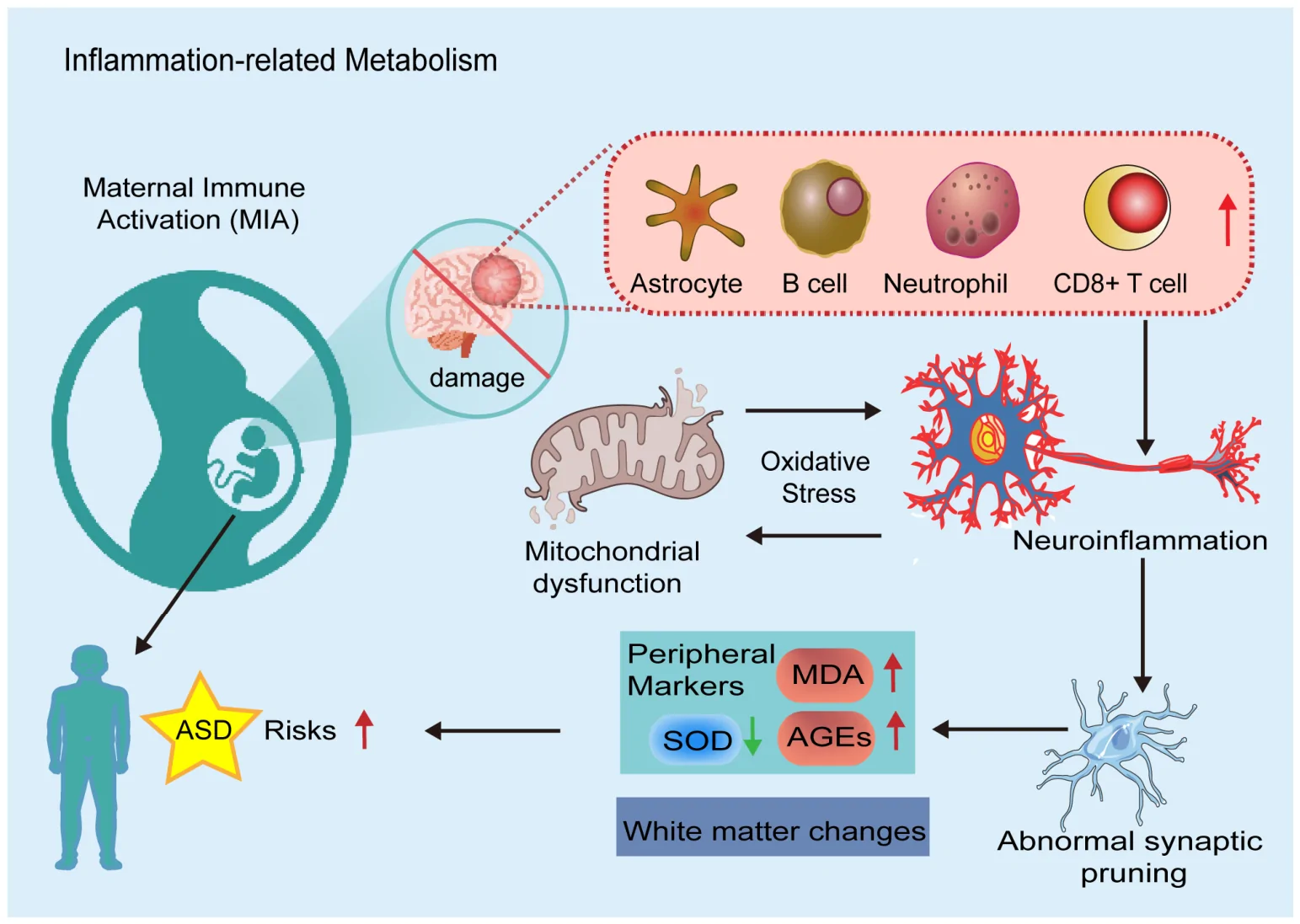
Inflammation-related metabolic dysregulation in ASD. This schematic illustrates the proposed cascade of inflammation-metabolism interactions in ASD pathogenesis. Maternal immune activation (MIA) during pregnancy is associated with a cycle of mitochondrial dysfunction and oxidative stress, potentially contributing to the dysregulation of peripheral metabolic biomarkers (elevated MDA and AGEs, decreased SOD activity), central neuroinflammation, proposed altered microglial synaptic pruning, and white matter changes, which collectively may modulate ASD risk. These dysregulated pathways represent potential targets for metabolite-based ASD interventions. Note: The translational perspectives depicted herein highlight critical methodological considerations (e.g., biomarker limitations and model heterogeneities) rather than absolute definitive causal relationships. Precise translational definitions have been referenced in the main text to address historical gaps in metabolic subtyping. The upward and downward arrows (↑/↓) denote an increase and decrease, respectively. Abbreviations: ASD = autism spectrum disorder; MIA = maternal immune activation; MDA = malondialdehyde; SOD = superoxide dismutase; AGEs = advanced glycation end products.

Representative studies have highlighted these characteristic interactions: Maternal immune activation, triggered by maternal infection during pregnancy, significantly increases the risk of ASD in offspring. Experimental models suggest that this elevated risk is closely linked to downstream neuroinflammation and oxidative stress metabolic disorders in the progeny [69]. Clinically, peripheral blood from children with ASD exhibits marked oxidative stress, evidenced by decreased antioxidant enzyme activity, increased lipid peroxidation, and the prominent accumulation of advanced glycation end-products [70,71]. Furthermore, the widespread mitochondrial dysfunction observed in patients with ASD increases intracellular free radical generation, which exacerbates central neuroinflammation and establishes a vicious cycle of combined immune and metabolic dysregulation [36] (Figure 4).

## 3. Metabolite-Based Interventions

### 3.1. Clinical Applications at the Human Level

At present, a variety of metabolite-based intervention methods have been applied in the clinical field (Table 2).

### 3.2. Interventions in Preclinical Animal Models

Similarly, metabolite-targeted interventions for ASD have been proven reliable and effective in pre-clinical animal models. We have compiled several that are of great research value and show much potential (Table 3).

### 3.3. Interventions Targeting Gut Microbiota-Derived Metabolites

#### 3.3.1. Clinical Research Progress

##### AB-2004

Building upon these findings, an open-label Phase 1b/2a clinical trial was conducted to evaluate the safety and efficacy of AB-2004, a microbial phenolic metabolite-targeting agent, in adolescent patients with ASD. The study enrolled 30 adolescents aged 12 to 16 years (29 males, 1 female) with ASD and GI symptoms, who received an 8-week course of dose-escalated AB-2004. Results indicated that AB-2004 reduced levels of 4-EPS and other ASD-associated microbial metabolites in both urine and serum, demonstrating that targeted binding of gut-derived microbial metabolites effectively decreases their systemic concentrations [14]. However, this trial has notable limitations. Its open-label, single-arm design lacks a placebo control, which cannot eliminate assessment bias and placebo effects, thereby weakening the robustness of the efficacy findings.

##### Probiotics, Prebiotics, and Synbiotics

Probiotics, prebiotics, and synbiotics are core intervention strategies targeting the gut microbiota and acting through the gut–brain axis [78]. By regulating the tryptophan metabolic pathway, such interventions can improve core social, emotional, and cognitive symptoms in individuals with ASD, offering synergistic benefits when combined with behavioral therapies [79]. Furthermore, by modulating the production of short-chain fatty acids (SCFAs), these treatments can repair intestinal barrier damage, inhibit inflammatory cascades, and concurrently alleviate gastrointestinal comorbidities and neurobehavioral symptoms [80]. However, existing studies are limited by small sample sizes and high heterogeneity, precluding high-level evidence-based recommendations. Significant individual differences in treatment response, an incomplete understanding of the dual role of SCFAs in ASD pathophysiology, a lack of long-term safety data, and the failure to identify specific responsive metabolic subtypes collectively hinder their clinical translation.

In fact, recent comprehensive reviews indicate that existing clinical trials on probiotic supplementation show inconsistent effects on the core social communication symptoms of children with ASD, and high-level evidence-based recommendations cannot be formed until large multi-center Randomized Controlled Trials (RCTs) are conducted [78,79]. The current body of evidence is fundamentally constrained by heterogeneous formulations, inconsistent intervention durations, and a lack of metabolic stratification. High-level, evidence-based clinical guidelines await the completion of large-scale, multicenter randomized controlled trials (RCTs).

#### 3.3.2. Preclinical Mechanism Validation

##### Indolepropionic Acid (IPA)

A recent preclinical investigation utilizing 16p11.2+/− murine models demonstrated that indolepropionic acid supplementation significantly ameliorated social and cognitive deficits while restoring inhibitory neurotransmitter homeostasis. Behavioral assessments revealed enhanced social interaction durations and spatial memory performance in 16p11.2+/− mice following IPA administration. Mechanistically, IPA attenuated hyperactivation of dentate gyrus (DG) neurons in the hippocampal formation, concomitant with increased gamma-aminobutyric acid synthesis. Molecular analyses identified reduced expression of Mitogen-Activated Protein Kinase 3 (MAPK3, located within the 16p11.2 locus) and its encoded protein extracellular signal-regulated kinase 1 (ERK1), including diminished phosphorylated ERK1 (p-ERK1) levels in the hippocampal DG region. Notably, IPA restored neuronal excitation-inhibition balance by augmenting ERK1 phosphorylation without altering MAPK3 transcriptional or translational output [18].

A primary limitation of this study is its exclusive reliance on a single murine model with a 16p11.2+/− heterozygous deletion. Because validation has not been extended to other genetic or environmentally induced models—nor to human subjects—there are significant limitations in extrapolating these therapeutic effects to the broader, highly heterogeneous clinical ASD population.

##### Butyrate

Hypotheses propose that maternal butyrate insufficiency during pregnancy could predispose offspring to neurodevelopmental vulnerabilities. Experimental validation was provided by a murine study demonstrating that maternal butyrate supplementation during gestation and lactation elevated offspring butyrate levels, thereby attenuating neurodevelopmental abnormalities and autism-like behaviors in genetically predisposed progeny [76]. The study mechanistically revealed that maternal-derived butyrate undergoes transplacental and transmammary transmission, directly influencing fetal and neonatal neurodevelopment. Prenatal butyrate administration restored histone deacetylase inhibition activity in offspring hippocampi, promoting synaptic plasticity through Brain-derived neurotrophic factor (BDNF)-TrkB signaling potentiation. Furthermore, butyrate-modulated shifts in maternal gut microbiota composition were associated with enhanced intestinal barrier function and reduced pro-inflammatory cytokine exposure in utero.

##### Bacteroides Fragilis (Human Commensal Bacterium)

In the context of environmental risk factors, the poly(I:C)-induced maternal immune activation (MIA) mouse model has been extensively utilized. Studies have demonstrated that MIA can lead to marked gut microbiota dysbiosis and elevated levels of serum 4-ethylphenylsulfate (4-EPS) in offspring [27]. Notably, microbial metabolic interventions, such as the administration of Bacteroides fragilis, have been shown to correct gut permeability and ameliorate anxiety-like and stereotypic behaviors in these specific models, suggesting a potential microbiome-mediated metabolic pathway for intervention.

##### Lactobacillus Reuteri (Probiotic Strain MM4-1A)

Regarding genetic susceptibility, metabolic interventions have also been explored in specific genetic knockout models, such as the Shank3 KO mice. Preclinical research indicates that metabolic modulation—for instance, through Lactobacillus reuteri-mediated oxytocin pathways—may effectively reverse impaired synaptic plasticity in the ventral tegmental area (VTA) and rescue social deficits in these animals [77]. These preclinical findings provide a necessary, albeit preliminary, foundational basis for evaluating metabolite-targeted strategies prior to clinical translation.

### 3.4. Interventions Targeting Mitochondrial Energy Metabolism

#### 3.4.1. Ketogenic Diet

The proposed mechanisms of the ketogenic diet involve modulation of energy metabolism, gut microbiota restructuring, and attenuation of neuroinflammatory processes. An open-label clinical trial involving 15 pediatric subjects with ASD evaluated a 3-month modified ketogenic gluten-free diet supplemented with medium-chain triglycerides (MCT) [73]. Following the intervention, participants demonstrated significant improvements in specific core autism features, particularly in the ADOS-2 total score and social affect subdomain, as well as several CARS-2 items (e.g., imitation and fear) [73].

However, no significant improvements were observed in restricted and repetitive behaviors. Furthermore, this dietary intervention faces practical challenges regarding adherence; the study reported a substantial drop-out and non-initiation rate among the recruited cohort [73]. Long-term ketogenic diet intervention in children may also carry potential risks including growth retardation, dyslipidemia, and gastrointestinal discomfort, requiring personalized nutritional assessment and monitoring during implementation [81].

#### 3.4.2. Acetyl-L-Carnitine

While clinical observations indicate significantly reduced intestinal acetate and butyrate levels in children with ASD compared to neurotypical controls [82], the mechanistic efficacy of acetyl-L-carnitine has predominantly been validated in preclinical models. A controlled study utilizing ASD model rats compared behavioral and biochemical outcomes between acetyl-L-carnitine-supplemented and non-supplemented cohorts. Behavioral phenotyping revealed that non-supplemented ASD rats exhibited diminished exploratory behaviors toward novel conspecifics and objects, coupled with increased self-grooming frequencies—phenotypic hallmarks of social withdrawal, restricted interests, and repetitive behaviors. Fecal metabolomic analyses reported markedly lower acetate and butyrate concentrations in non-supplemented ASD rats relative to healthy controls [17].

Mechanistic investigations propose that acetyl-L-carnitine enhances microbial synthesis of SCFAs, which in turn reinforces intestinal barrier integrity through tight junction protein upregulation (e.g., occludin, zonula occludens-1) and attenuates neuroinflammation via microglial polarization toward anti-inflammatory phenotypes [83]. These findings underscore the therapeutic potential of acetyl-L-carnitine as a microbiota-metabolism modulator for dual targeting of GI and neurobehavioral symptoms in ASD. Further research is required to delineate dose–response relationships and evaluate translational applicability in human clinical populations.

### 3.5. Interventions Targeting Immune–Metabolic Interactions

#### 3.5.1. Suramin

Suramin, one of the oldest synthetic therapeutic agents in clinical use, is recognized for its well-characterized activity as an inhibitor of purinergic signaling. The Suramin Autism Treatment-1 (SAT-1) trial was a placebo-controlled clinical trial, designed to evaluate the safety and biological activity of a single-dose suramin intervention versus placebo. A total of 10 children diagnosed with ASD were enrolled in the study cohort. Following suramin administration, transient improvements in linguistic, social, behavioral, and developmental domains were observed, with maximal therapeutic effects persisting for 3 weeks before gradual decline to baseline levels over the subsequent 3-week period [72]. The observed temporal correlation between therapeutic benefit attenuation and pharmacokinetic clearance underscores the transient nature of single-dose efficacy. This pharmacodynamic profile necessitates further investigation into optimized dosing regimens.

#### 3.5.2. N-Acetylcysteine (NAC)

N-acetylcysteine (NAC), a glutathione precursor and potential glutamatergic modulator, has been evaluated in preliminary clinical settings to target oxidative stress and redox imbalance. A pilot randomized controlled trial demonstrated that the administration of oral NAC was associated with significant reductions in irritability among children with ASD. However, the preliminary nature of these findings, coupled with a reliance on informant-based behavioral scales and concurrent psychotropic medication use, necessitates further validation in larger cohorts [74].

#### 3.5.3. Sulforaphane

Similarly targeting immune–metabolic interactions, sulforaphane—a dietary phytochemical—is hypothesized to up-regulate the Keap1-Nrf2 cytoprotective signaling pathway, thereby mitigating oxidative stress and neuroinflammation. A randomized, double-blind, placebo-controlled trial reported that sulforaphane treatment was associated with improvements in social responsiveness and aberrant behaviors in young men with moderate to severe ASD. Notably, these behavioral gains were observed to regress upon treatment cessation. Current evidence remains constrained by restricted demographic representation (exclusively male cohort) and potential selection bias [75].

## 4. Clinical Translation Perspectives of Metabolite-Targeted Interventions

### 4.1. Classification of Translational Priority Levels

The preceding sections have systematically mapped the targetable metabolic abnormalities in ASD alongside current preclinical and clinical evidence for metabolite-targeted interventions. Despite the pathological correlations discussed above, translating metabolite-targeted interventions into clinical practice faces a significant gap. To date, no pharmacological treatments currently address the core deficits of ASD, and interventions have primarily targeted secondary symptoms [84]. Very few metabolic interventions have advanced to formal, large-scale phase III clinical trials.

To clarify the translational trajectory of different interventions and optimize the allocation of research resources, we propose an adapted translational priority grading framework informed by the core principles of the Grading of Recommendations Assessment, Development and Evaluation (GRADE) system [85]. In accordance with GRADE methodology, priority levels in this framework were systematically downgraded due to prevalent study limitations (e.g., single-arm open-label designs, inadequate cohort sizes) and indirectness of evidence (e.g., exclusive reliance on single-gene animal models without human validation). Conversely, high priority is strictly reserved for interventions supported by high-quality evidence, such as well-designed multi-center randomized controlled trials demonstrating precise target engagement.

High-priority interventions warrant immediate clinical translation. Key candidates include the ketogenic diet and AB-2004. The ketogenic diet is already in routine clinical use for individuals with ASD and comorbid epilepsy. Supported by prospective clinical trials, it targets mitochondrial energy metabolism and benefits from high clinical accessibility; notably, the mitochondrial dysfunction subtype has been identified as its core responsive population. While the ketogenic diet is frequently discussed as a metabolic intervention, it is imperative to clarify that its long-term clinical safety and tolerability data are primarily extrapolated from pediatric epilepsy cohorts [84]. Consequently, its long-term safety profile specifically within pure ASD populations remains insufficiently validated and necessitates further longitudinal investigation. AB-2004 is the leading candidate targeting ASD-specific microbial metabolites, having completed phase 1b/2a trials. With a well-defined target (4-EPS) and verifiable pharmacodynamic biomarkers, preliminary data suggest it significantly ameliorates core behavioral symptoms and gastrointestinal comorbidities, positioning it as a potential first-in-class FDA-approved metabolite therapy for ASD.

Medium-priority interventions require further clinical validation to strengthen their evidence base. These include probiotics/prebiotics/synbiotics, acetyl-L-carnitine, suramin, and minocycline adjunctive therapy. While these approaches possess clear metabolic regulatory mechanisms, favorable overall safety profiles, and positive preliminary RCT data in pediatric ASD, current evidence remains constrained. Key limitations include small cohort sizes, significant methodological heterogeneity, and the absence of validated predictive biomarkers for patient stratification. Large-scale, multicenter RCTs are imperative to substantiate their clinical benefits and precisely define responsive subpopulations.

Low-priority interventions—such as indolepropionic acid (IPA), butyrate supplementation, and oral vancomycin—require deepening preclinical mechanistic research prior to cautious clinical exploration. These strategies either rely on efficacy data from single animal models without human clinical trial support, or carry significant safety risks (e.g., vancomycin-induced antimicrobial resistance) and uncertain target specificity. Given the high translational risk, rigorous validation across diverse multi-model systems and the comprehensive elucidation of causal mechanisms are prerequisites for future clinical trials.

### 4.2. Core Barriers

Despite the therapeutic potential of metabolite-targeted interventions, the clinical translation process remains exceptionally slow, restricted primarily by four core barriers:

#### 4.2.1. Translational Gap Between Preclinical Models and Human ASD

The vast majority of preclinical studies utilize single-gene or single-environment-induced ASD models characterized by high homogeneity. However, preclinical translation is frequently challenged by the profound behavioral and clinical heterogeneity of human ASD, which represents a major hurdle when extrapolating therapeutic outcomes from single-gene rodent models [86]. Furthermore, preclinical interventions typically target critical windows of early neurodevelopment, whereas clinical trials predominantly enroll children (aged 3–12 years) with established neurodevelopmental abnormalities. This temporal disconnect frequently leads to the dilemma of interventions succeeding in animal models but failing in human trials.

#### 4.2.2. Lack of Validated Predictive and Pharmacodynamic Biomarkers

While AB-2004 utilizes 4-EPS as a pharmacodynamic biomarker, the majority of other therapeutic strategies currently lack broadly validated markers. The absence of indicators to prescreen responsive populations results in the inclusion of non-responsive subjects in clinical trials, thereby diluting overall efficacy signals. Additionally, the current reliance on non-standardized or subjective observational measures frequently fails to accurately capture the modulatory effects of interventions; thus, there is a critical need for quantifiable, reproducible biomarkers and surrogate endpoints to stratify biological subtypes and objectively assess treatment efficacy [87].

#### 4.2.3. Insufficient Stratification of ASD Metabolic Subtypes

Most existing clinical trials employ a “one-size-fits-all” strategy for the general ASD population, neglecting stratification based on metabolic profiles. For instance, the ketogenic diet primarily benefits the mitochondrial dysfunction subtype, while probiotics specifically target gut microbiota-derived metabolic dysregulation. The failure to stratify cohorts is a primary factor contributing to the inconsistent efficacy observed across current studies. Previous attempts to identify metabolic biomarkers or subtypes have frequently treated ASD cohorts as biochemically homogeneous, yielding results that are difficult to replicate due to small sample sizes, unmeasured environmental confounders (e.g., diet, medications), and profound interindividual heterogeneity [88]. To address this conceptual gap, and drawing upon the diverse metabolic dysregulations reviewed, we propose a hypothetical conceptual model consisting of four metabolic categories: gastrointestinal microbiota metabolism, energy/mitochondrial metabolism, neurotransmitter metabolism, and immunometabolic interactions. It must be explicitly acknowledged that these categories represent a theoretical framework rather than established clinical subtypes. Currently, ASD encompasses a highly heterogeneous spectrum, and there is insufficient evidence from large-scale clinical clustering trials to support the definitive stratification of patients into exactly these four subtypes. This lack of empirical clinical clustering validation remains a major limitation in the current field, highlighting a critical direction for future precision medicine research.

#### 4.2.4. Methodological Limitations in Clinical Trials

Many existing trials are hampered by small sample sizes, single-center designs, a lack of randomized double-blind placebo controls, short follow-up durations, and inconsistent primary endpoints. These methodological limitations diminish the robustness of the findings, precluding the formulation of high-level evidence-based guidelines for broad clinical implementation.

### 4.3. Strategies to Break Through Clinical Translation Barriers

To bridge the translational gap, future preclinical studies must adopt multi-model validation systems (incorporating genetic, environmental, and humanized models) that more accurately reflect the heterogeneity of human ASD. Aligning preclinical intervention windows and behavioral evaluation metrics with those used in clinical trials will significantly improve translational predictability.

To address the biomarker deficit, large-scale prospective multi-omics cohort studies are essential to identify and validate predictive markers of treatment response and pharmacodynamic engagement, ultimately establishing standardized profiling protocols for metabolic biomarkers in ASD.

To overcome insufficient stratification, future clinical trials should implement a “biomarker-driven stratification design.” For instance, stratifying patients based on baseline metabolic profiles (such as high-density lipoprotein or albumin levels) or specific behavioral severity thresholds may help identify distinct subpopulations more likely to respond to targeted interventions. This tailored approach could potentially mitigate the masking of therapeutic effects caused by the inherent clinical heterogeneity of ASD.

To resolve methodological limitations, researchers should prioritize multicenter, large-cohort, randomized double-blind placebo-controlled trials. Utilizing established primary efficacy measures validated in large-scale RCTs—such as the Aberrant Behavior Checklist Irritability (ABC-I) subscale—and extending follow-up periods will be crucial for rigorously evaluating the long-term efficacy and safety of these interventions [89].

## 5. Conclusions and Future Directions

In summary, metabolic dysregulation represents a promising and biologically relevant perspective for understanding the pathogenesis and intervention of ASD. Accumulating evidence suggests that alterations in gut microbiota-derived metabolites, energy metabolism, neurotransmitter pathways, and immune–metabolic interactions may contribute to ASD development by broadly influencing neurodevelopmental processes.

Despite broad therapeutic potential, the clinical translation of metabolite-targeted interventions for ASD remains challenged by methodological hurdles and phenotypic heterogeneity. To advance the field, future research must prioritize three core objectives: first, initiating large-scale prospective multi-omics cohort studies to establish causal relationships and validate reliable metabolic biomarkers; second, conducting rigorously designed, multicenter randomized controlled trials that utilize biomarker-guided metabolic stratification; and third, developing a standardized classification system for ASD metabolic subtypes. By clarifying the mechanistic links between metabolism and neurodevelopment, these targeted strategies will facilitate the critical transition from symptom-based management to precision, mechanism-driven therapies, ultimately improving long-term neurodevelopmental outcomes and the quality of life for individuals with ASD.

## Figures and Tables

**Table 1:** Summary of metabolic abnormalities reported in ASD.

Metabolic Pathway	Key Metabolite/Biomarker	Study Type	Population/Sample	Major Findings	Biological Implication	Evidence Level	References
Gut microbiota-derived metabolism	4-ethylphenyl sulfate (4-EPS)	Human metabolomics + animal validation	Children with ASD cohorts and mouse models	Elevated circulating 4-EPS levels associated with ASD behavioral phenotypes	Microbial metabolites may affect brain myelination and anxiety-like behaviors	Human observational + animal validation	[30,31]
	Indole 3-propionic acid (IPA)	Animal validation & Human observational	IUGR children and rat models	Reduced serum IPA levels; IPA supplementation restores ASD-like symptoms and hippocampal lesions	IPA acts as a protective microbiota-derived metabolite via AHR signaling	Animal validation	[32]
	Indole-3-acetic acid (IAA)/Tryptophan	Metabolomics and microbiome analysis	ASD pediatric cohorts	Altered tryptophan to indole-derived metabolite ratios (e.g., TRP/IAA) correlating with symptom severity	Gut microbiota influence host tryptophan metabolism and neurodevelopment	Human observational	[33]
Energy metabolism	Brain lactate, Mitochondrial Complex I	In vivo Neuroimaging (MRS and PET)	Children and adults with ASD	Elevated brain lactate doublets and lower availability of mitochondrial complex I in specific brain regions	Direct in vivo evidence of impaired mitochondrial energy production in the ASD brain	Human observational	[34,35]
	ATP, ROS, Calcium (Ca^2+^)	Comprehensive review	ASD patient cohorts	Aberrant ATP production, Ca^2+^ homeostasis, and ROS accumulation negatively impact brain development	Supports mitochondrial dysfunction hypothesis in ASD	Review	[36]
	Carnitine, lactate, pyruvate	Systematic review and meta-analysis	children with ASD	High prevalence of abnormal mitochondrial biomarkers (carnitine, lactate) correlating with ASD severity	Impaired fatty acid β-oxidation and mitochondrial metabolism	Meta-analysis	[37]
Neurotransmitter metabolism	Serotonin	Systematic review and meta-analysis	ASD individuals	Elevated whole blood and platelet-rich plasma serotonin levels in ~28% of ASD individuals	Altered serotonergic signaling may contribute to ASD pathology	Meta-analysis	[38]
	Kynurenine (KYN) pathway metabolites	Meta-analysis & Observational profiling	ASD pediatric populations	Recent meta-analyses find no significant peripheral blood KYN abnormalities, though urinary indices may co-vary with immune markers	Peripheral KYN alterations may not be primary drivers; urinary profiling reflects localized immune–metabolic cross-talk	Meta-analysis & Human observational	[39,40]
	Malondialdehyde (MDA), Superoxide dismutase (SOD)/GPx1 activity	Biochemical assays	ASD pediatric cohorts and maternal diabetes rat models	Decreased antioxidant enzyme activity (GPx1, SOD2) and elevated oxidative stress markers	Redox imbalance and suppressed SOD2 expression contribute to neurodevelopmental dysfunction	Human observational & Animal validation	[41,42]
Immune–metabolic interactions	Pro-inflammatory cytokines (e.g., IL-1β, IL-6, IFN-γ)	Systematic review and meta-analysis	Individuals with ASD	Significantly elevated concentrations of pro-inflammatory cytokines compared to healthy controls	Inflammatory signals and abnormal cytokine profiles dominate the ASD immune phenotype	Meta-analysis	[43]

Abbreviations: ASD, autism spectrum disorder; IUGR, intrauterine growth restriction; AHR, aryl hydrocarbon receptor; TRP, tryptophan; MRS, magnetic resonance spectroscopy; PET, positron emission tomography; ATP, adenosine triphosphate; ROS, reactive oxygen species; GPx1, glutathione peroxidase 1; IL-1β, interleukin-1 beta; IL-6, interleukin-6; IFN-γ, interferon gamma.

**Table 2:** Clinical studies of metabolite-targeted interventions in ASD.

Intervention	Study Design	Participants (*n*)	Age (Years)	Gender (Male/Female)	Target Mechanism	Clinical Outcomes	Evidence Level	Key Limitations	References
AB-2004	Open-label phase 1b/2a trial	30	12–17	29/1	Adsorption of gut microbial metabolites (e.g., aromatic compounds, 4-EPS)	Circulating microbial metabolites ↓, anxiety ↓, irritability ↓, GI symptoms ↓	Early clinical evidence	Small sample, no placebo control	[14]
Suramin	Double-blind randomized pilot trial	10	5–14	10/0	Purinergic signaling inhibition, cell danger response normalization	Language ↑, social interaction ↑, repetitive behaviors ↓	Randomized pilot clinical evidence	Very small cohort, single-dose treatment	[72]
Modified ketogenic gluten-free diet with MCT	Open-label clinical trial	15	3–13	13/2	Modulation of neural cellular metabolism (ketone utilization), metabolic/inflammatory marker alteration	Improved ADOS-2 total and social affect scores; improved CARS-2 scores (imitation, body use, fear)	Open-label clinical trial evidence	Small sample size, open-label (no control/placebo group), high drop-out rate	[73]
Probiotics supplementation	Clinical trial	40	2–5	NR	Modulation of gut microbiota and dysbiosis	Significant increase in beneficial bacteria, anxiety reduction, and improved sleep quality	Clinical trial evidence	Small sample size, short intervention duration	[16]
Oral N-acetylcysteine (NAC)	Double-blind, randomized, placebo-controlled pilot trial	33	3.2–10.7	31/2	Potential glutamatergic modulation and antioxidant support (via acting as a glutathione precursor)	Associated with significant reductions in irritability (ABC-Irritability subscale) and a trend toward improved stereotypic behaviors.	Pilot RCT	Small sample size, relatively high attrition rate (25%), concurrent psychotropic medication use, and reliance on informant-based behavioral scales without direct in vivo biomarker validation.	[74]
Sulforaphane (phytochemical from broccoli sprout extracts)	Double-blind, randomized, placebo-controlled trial	44	13–27	44/0	Hypothesized to up-regulate cellular stress responses (e.g., Keap1-Nrf2 cytoprotective signaling pathway) to mitigate oxidative stress and neuroinflammation.	Associated with significant improvements in social responsiveness (SRS) and aberrant behaviors (ABC), which regressed upon treatment cessation.	RCT	Single-site study, exclusively male and predominantly Caucasian cohort, and potential selection bias (unusually high prevalence of ‘fever responders’ at 80%).	[75]

NR: Not Reported in the publicly accessible abstract. Complete demographic data were inaccessible due to publication paywall restrictions, and missing values were honestly retained to strictly prevent unverified data imputation. The upward and downward arrows (↑/↓) denote an increase and decrease, respectively. Abbreviations: ASD, autism spectrum disorder; 4-EPS, 4-ethylphenyl sulfate; GI, gastrointestinal; MCT, medium-chain triglycerides; ADOS-2, Autism Diagnostic Observation Schedule, Second Edition; CARS-2, Childhood Autism Rating Scale, Second Edition; ABC, Aberrant Behavior Checklist; RCT, randomized controlled trial; SRS, Social Responsiveness Scale; Keap1, Kelch-like ECH-associated protein 1; Nrf2, nuclear factor erythroid 2-related factor 2.

**Table 3:** Metabolite-targeted interventions in ASD animal models.

Intervention	Study Design	Model	Dose/Duration	Target Mechanism	Behavioral Outcomes	Evidence Level	Key Limitations	References
Indolepropionic acid (IPA)	Experimental intervention	16p11.2+/− ASD mouse	20 mg/kg, ~2 weeks	Mitogen-Activated Protein Kinase(MAPK) signaling modulation, inhibitory neurotransmission ↑	Social interaction ↑, cognitive performance ↑	Preclinical evidence	Single genetic model	[18]
Butyrate supplementation	Maternal dietary supplementation	ASD offspring mouse model	~30 mg/kg gestation/lactation	Histone deacetylase(HDAC) inhibition, synaptic plasticity ↑	Autism-like behaviors ↓ in offspring	Preclinical evidence	Maternal exposure model	[76]
Acetyl-L-carnitine	Pharmacological intervention	ASD rat model	~100 mg/kg, 28 days	Mitochondrial metabolism ↑, fatty acid oxidation ↑	Social behavior ↑, stereotypy ↓	Preclinical evidence	Limited behavioral assays	[17]
Bacteroides fragilis (human commensal bacterium)	Preclinical experimental intervention	Maternal immune activation (MIA) mouse model	~1 × 10^10^ CFU, oral gavage every other day for 6 days at weaning	Restoration of intestinal barrier integrity (claudins, ZO-1), reduction in colonic IL-6, and modulation of circulating metabolites (e.g., serum 4-EPS reduction)	Ameliorated communicative, stereotypic, and anxiety-like behaviors (deficits in sociability remained unrescued)	Preclinical evidence	Single environmental etiology model, non-persistent bacterial colonization, and failure to rescue core social interaction deficits.	[27]
Lactobacillus reuteri (probiotic strain MM4-1A)	Preclinical experimental intervention	Shank3B−/− knockout (KO) genetic mouse model	~1 × 10^8^ organisms/day in drinking water, 4 weeks	Vagus nerve-dependent upregulation of oxytocin in the paraventricular nucleus (PVN) and restoration of social interaction-induced synaptic plasticity (LTP) in VTA dopaminergic neurons	Rescued sociability and reciprocal social interaction deficits (hypoactivity remained uncorrected)	Preclinical evidence	Tested exclusively in male mice, strictly dependent on intact vagus nerve and oxytocin receptor signaling, with no improvement in non-social motor deficits.	[77]

The upward and downward arrows (↑/↓) denote an increase and decrease, respectively. Abbreviations: ASD, autism spectrum disorder; CFU, colony-forming unit; IL-6, interleukin-6; 4-EPS, 4-ethylphenyl sulfate; LTP, long-term potentiation; VTA, ventral tegmental area.

## Data Availability

No new data were created or analyzed in this study. Data sharing is not applicable to this article.
